# Case report: Bilateral facial palsy with paresthesias and positive anti-GT1a antibodies

**DOI:** 10.3389/fimmu.2024.1410634

**Published:** 2024-06-07

**Authors:** Xiaoxuan Xu, Zhihan Wang, Chang Su, Lin Cong, Dongming Zheng

**Affiliations:** Department of Neurology, Shengjing Hospital of China Medical University, Shenyang, China

**Keywords:** GBS, Guillain-Barré syndrome, variant, bilateral facial palsy, anti-gangliosides antibodies

## Abstract

Bilateral facial palsy with paresthesia (FDP) is a rare variant of GBS, characterized by simultaneous bilateral facial palsy and paresthesia of the distal limbs. Mounting evidence indicates that the presence of anti-GT1a IgG has a pathogenic role as an effector molecule in the development of cranial nerve palsies in certain patients with GBS, whereas anti-GT1a antibody is rarely presented positive in FDP. Here, we report the case of a 33-year-old male diagnosed with FDP presented with acute onset of bilateral facial palsy and slight paresthesias at the feet as the only neurological manifestation. An antecedent infection with no identifiable reason for the fever or skin eruptions was noted in the patient. He also exhibited cerebrospinal fluid albuminocytologic dissociation and abnormal nerve conduction studies. Notably, the testing of specific serum anti-gangliosides showed positive anti-GT1a IgG/IgM Ab. The patient responded well to intravenous immunoglobulin therapy. This case brings awareness to a rare variant of GBS, and provides the first indication that anti-GT1a antibodies play a causative role in the development of FDP. The case also suggests that prompt management with IVIG should be implemented if FDP is diagnosed.

## 1 Introduction

Bilateral facial palsy with paresthesias (FDP) is a rare variant of Guillain-Barré syndrome (GBS), with an incidence of less than 1% among all GBS cases (1). This particular GBS variant is characterized by pronounced bilateral facial weakness, paresthesias in the distal extremities, diminished tendon reflexes, and the absence of ophthalmoplegia, ataxia, or limb weakness. In previous limited case reports of this variant, antiganglioside antibody testing predominantly yielded negative results, with the exception of a minority presenting with positive anti-GM2 antibodies (2). Further investigation of the antiganglioside antibodies associated with this unique GBS variant is warranted, given the established association between some GBS variants and specific antiganglioside antibodies due to the disproportionate enrichment of target glycolipids in different nerves (3). Testing of antiganglioside antibodies is helpful for diagnosis of GBS and its variants.

The current report presents the case of a young male patient who exhibited typical clinical manifestations, alongside cerebrospinal fluid (CSF) analysis and electrophysiological results, collectively suggesting a diagnosis of bilateral facial paralysis with paresthesias. Notably, this patient tested positive for both anti-GT1a IgM and IgG antibodies, a finding not previously reported in the literature. This case may contribute a novel perspective to the existing knowledge of rare GBS variants, potentially informing future diagnostic and therapeutic approaches.

## 2 Case report

A 33-year-old male with known co-morbid hypertension and type 2 diabetes mellitus was admitted to our hospital due to an acute onset of left facial weakness persisting for a week, followed by right facial weakness lasting for three days. He reported having a fever and skin eruptions a week prior to onset of illness. Initially, the patient believed these symptoms were indicative of the flu and self-administered acetaminophen for two days without consulting a healthcare professional. Shortly afterwards, the fever subsided, and the skin eruptions resolved. Subsequently, he developed a flattened left nasolabial fold, lack of wrinkling of the left forehead and incomplete closure of the left eye. Four days later, he noticed the same kind of facial muscle weakness in the right side of his face, resulting in an inability to completely close both eyes and drooping of the corners of his mouth. Approximately three days after the onset of right facial paralysis, he was referred to our hospital for further valuation.

The patient denied experiencing symptoms including diplopia, facial numbness, tinnitus, dysphagia, or neck stiffness or weakness. No numbness, pain, or weakness elsewhere in his body were reported. He had no history of head or neck trauma or recent travel. His most recent vaccination was the COVID-19 vaccine, which was administered three years ago.

On general physical examination, no significant abnormalities were found. Blood pressure was 150/80 mmHg, his pulse was 75 beats per minute, and his body temperature was 36.7°C. The patient’s heart rate and rhythm were regular with no murmurs. The breath sounds were clear to auscultation bilaterally with no wheezing, or rhonchi. The abdomen was soft, non-distended, and non-tender, with no palpable masses or organomegaly. On neurological examination, the patient was fully awake, alert and oriented to person, place and time. The pupils were equal, round and reactive to light and accommodation. Extraocular movements were intact in all directions, with smooth and coordinated pursuit. The patient exhibited normal face sensation across all three divisions of the trigeminal nerve. Temporomandibular joint movements were normal, and the patient exhibited strong jaw clench. However, the patient exhibits bilateral peripheral facial palsy. Facial expression movements and the ability to close the eyelids were both affected, making it difficult for him to perform actions such as frowning, blinking, smiling, and showing his teeth. The patient’s palate elevates symmetrically. Gag reflex is present. Tongue protrudes in the midline. No tongue atrophy or fasciculations present. The patient’s motor strength was 5/5 in bilateral upper and lower extremities. Muscle tone was normal in all four extremities. The patient’s gait was normal and there was no ataxia noted on finger to nose and heel to shin testing. Sensory examination revealed normal and symmetrical fine touch, pain, temperature, vibration and position senses. Deep tendon reflexes were 2+ in the upper extremities and 1+ in the lower extremities. The plantar reflexes were flexor bilaterally.

Serum analyses for liver, renal, and thyroid functions, cardiac enzymes, folic acid, and vitamin B12 were within normal limits. However, the fasting serum glucose level was elevated at 10.21 mmol/L. Serological tests for *treponema pallidum*, human immunodeficiency virus (HIV), hepatitis B virus (HBV), hepatitis C virus (HCV), herpes simplex virus (HSV), varicella-zoster virus (VZV), and *Mycoplasma pneumoniae* were negative. The patient’s head MRI, including the diffusion weighted imaging (DWI) sequence, revealed no abnormalities.

Cerebrospinal fluid (CSF) showed albuminocytological dissociation (opening pressure: 160 mm H2O, white blood cell count: 4 ×10^6^/L, protein concentration 0.68 g/L, and normal glucose and chloride levels). CSF culture for bacteria was negative. The serum was negative for ganglioside antibodies including IgG and IgM antibodies of GM1, GM2, GM3, GD1a, GD1b, GD3, GT1b, GQ1b, galactocerebroside and GalNAc-GD1a, but positive for GT1a IgG and lgM antibodies. Electromyography (EMG) demonstrated axonal degeneration in the bilateral facial nerves and mild damage to the sensory and motor fibers of peripheral nerves in the limbs.

**Figure 1:**
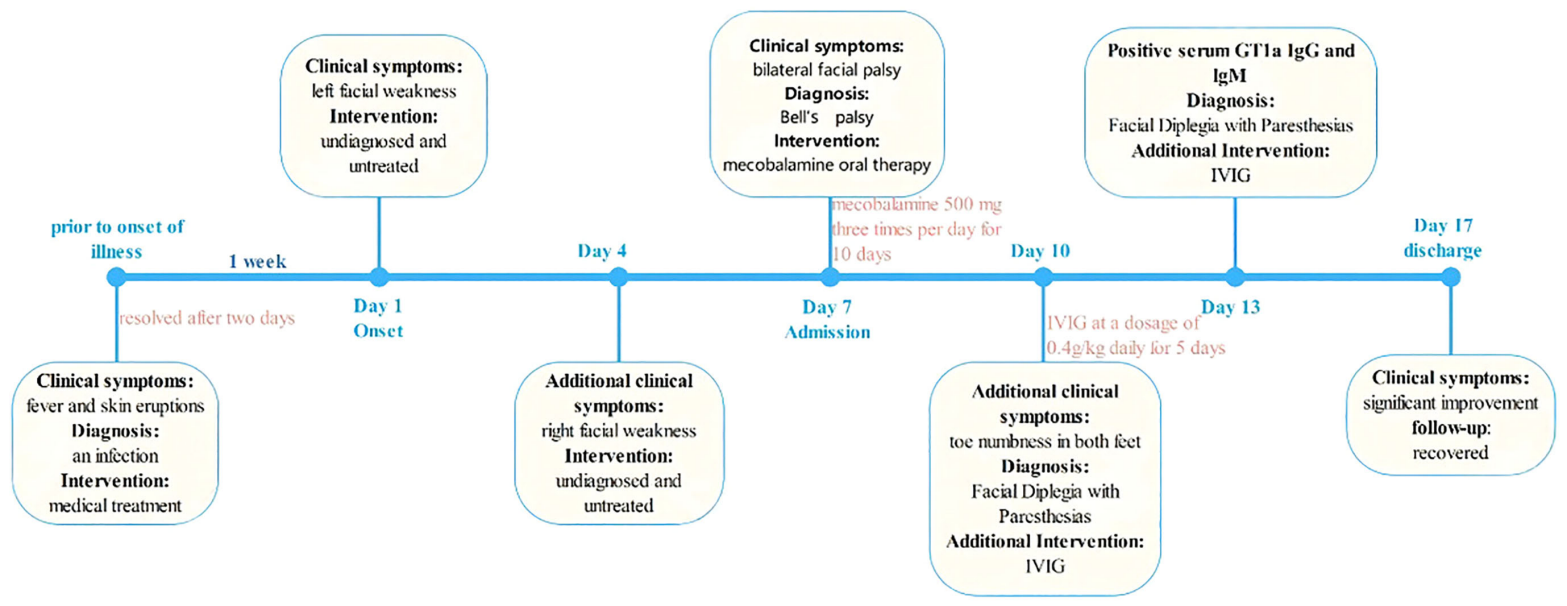
Timeline of the patient’s episodes and interventions.

On the third day of hospitalization, the patient reported toe numbness in both feet. Given the patient’s medical history, clinical manifestations, and laboratory findings, a diagnosis of bilateral facial palsy with paresthesias was considered. Treatment with intravenous immunoglobulin (IVIG) at a dosage of 0.4g/kg daily for 5 days was initiated. Additionally, oral mecobalamine was prescribed at a dose of 500 mg three times per day for ten days. The patient was discharged 10 days post-admission, showing complete resolution of toe numbness and significant improvement in bilateral facial paralysis. The timeline of the patient’s episodes and interventions is shown in **Figure 1**.

## 3 Discussion

Bilateral facial palsy is a rare clinical manifestation, with an annual incidence of only 1 per 5,000,000 individuals (4), and represents a mere 0.3%-2% of facial palsy cases (5). In contrast to unilateral facial paralysis, predominantly linked to Bell’s palsy, the etiology of bilateral facial paralysis is varied. A comprehensive study involving 326 patients with bilateral facial paralysis (6) identified Guillain-Barré syndrome (GBS) as the leading cause (27.6%), followed by Bell’s palsy (15.3%), and Lyme disease caused by Borrelia burgdorferi (13.2%). Other notable causes included chronic inflammatory polyneuropathy, HIV infection, Miller-Fisher syndrome, granulomatosis with polyangiitis, head trauma, leukemia, lymphoma, among others. However, in the research conducted by Gaudin et al., which encapsulated 13 years of clinical observations, Bell’s palsy was determined to be the most frequent diagnosis (35%), with GBS accounting for only 2% (7). Given the evidence indicating that GBS with facial palsy is frequently misdiagnosed as Bell’s palsy (8), it is possible that the prevalence of bilateral facial palsy attributable to GBS is significantly underestimated. Particularly, patients presenting with bilateral facial palsy accompanied by mild limb symptoms, such as in the case described herein, are prone to being erroneously diagnosed with Bell’s palsy.

Facial nerve paralysis is a common manifestation in GBS, with half of the affected individuals exhibiting bilateral involvement (9), often accompanied by limb weakness or paralysis of other cranial nerves. Isolated facial diplegia, without limb weakness, is exceedingly rare in GBS, with only a handful of cases reported thus far. In 1994, Ropper (10) was the first to document four cases presenting with acute, symmetrical bilateral facial weakness and numbness in the extremities, suggesting this condition might represent a regional variant of GBS, which he termed “Facial Diplegia with Paresthesias” (FDP). Subsequently, in 2009, Susuki et al. (2) delineated the clinical features of this GBS variant in a study involving 22 patients. Their findings indicated that FDP is typified by acute bilateral facial weakness, distal limb paresthesias, and diminished tendon reflexes, with few additional symptoms. The disorder frequently occurs post-infection, with 86% of patients reporting antecedent infectious symptoms. Albuminocytologic dissociation in cerebrospinal fluid is observed in all cases, and nerve conduction studies reveal limb demyelination in 64% of instances. Over time, this rare GBS variant has gained recognition and has been incorporated into various expert consensus statements and guidelines for GBS (11, 12). In recent years, particularly in the context of widespread COVID-19 vaccination, there has been a notable increase in GBS cases linked to vaccination (13). Among these, several instances of FDP associated with COVID-19 vaccination have been reported (14, 15). The prognosis for patients with FDP, whether related to vaccination or post-infectious as in our case, is generally favorable.

The current case is fully in line with the characteristics of FDP (16): bilateral paralysis in a short period of time, a history of prodromal infection, no limb weakness and other cranial nerve paralysis, absence of the limb tendon reflex, and numbness of both feet during hospitalization. Results from CSF examination and EMG also supported GBS. The current case reminds us again that a thorough history taken and neurological examination are crucial in making the correct diagnosis, as the patient was initially diagnosed with Bell’s palsy.

It is worth noting that our patient tested positive for anti-GT1a antibodies, a finding not previously reported in FDP patients. Testing for anti-ganglioside antibodies is instrumental in diagnosing GBS and its variants. Key anti-ganglioside antibodies linked to GBS include GM1, GD1a, GalNAc-GD1a, GM1b, GD3, CD1b, GT1a, and GQ1b. While routine anti-ganglioside antibody testing may not be critical for most patients with classical motor-sensory GBS, it holds significant diagnostic value for specific variants (12). For instance, anti-GQ1b antibody testing is recommended when Miller Fisher Syndrome (MFS) is suspected. To date, no specific anti-ganglioside antibodies have been definitively associated with bilateral facial palsy with paresthesias. Among the scant case reports on this variant, most showed no positive antibodies, with occasional reports of anti-GM2 (2) or anti-GD1a antibodies (17). The pathogenic role of anti-GT1a antibodies is only partially understood. These antibodies are closely associated with pharyngeal-cervical-brachial syndrome and are sometimes also present in Miller-Fisher syndrome (18). This association is likely due to the higher expression of GT1a in the cranial nerves, particularly the glossopharyngeal and vagal nerves. However, research by Koga et al. (19) has shown that, in addition to ophthalmoparesis and bulbar palsy, facial palsy is also common in patients with anti-GT1a IgG (ophthalmoparesis 57%, facial palsy 57%, bulbar palsy 70%). This suggests that the high expression of GT1a is not limited to the glossopharyngeal and vagal nerves. Anti-GT1a antibodies may bind to the nodal axolemma or neuromuscular junctions of peripheral nerves, leading to complement activation and damage to voltage-gated sodium channel clusters, ultimately disrupting axo-glial or neuromuscular junctions (20). Our findings suggest that anti-GT1a IgG may also play a pathogenic role in bilateral facial palsy with paresthesia.

The management of GBS encompasses supportive care alongside disease-modifying therapies such as IVIG or plasma exchange (PE). The optimal strategy for treating the diverse variants of GBS remains to be conclusively determined; however, the current practices for GBS treatment are deemed appropriate. Given that approximately 39% of patients with anti-GT1a IgG may require mechanical ventilation (19), patients should be closely monitored even if they do not have limb weakness, bulbar palsy, or respiratory failure.

## 4 Conclusions

Bilateral facial palsy with paresthesia is a rare variant of GBS. Comprehensive medical history, meticulous neurological examination, and supportive diagnostic tests are paramount in establishing the diagnosis and distinguishing this condition from other causes of bilateral facial palsy. The association between anti-GT1a antibodies and this specific variant of GBS merits further exploration through studies involving larger cohorts.

## Data availability statement

The original contributions presented in the study are included in the article/supplementary material. Further inquiries can be directed to the corresponding author.

## Ethics statement

The studies involving humans were approved by Medical Ethics Committee of Shengjing Hospital of China Medical University. The studies were conducted in accordance with the local legislation and institutional requirements. The participants provided their written informed consent to participate in this study. Written informed consent was obtained from the individual(s) for the publication of any potentially identifiable images or data included in this article.

## Author contributions

XX: Conceptualization, Writing – original draft, Writing – review & editing, Investigation. ZW: Writing – original draft, Conceptualization, Investigation. CS: Writing – original draft, Conceptualization, Investigation. LC: Writing – original draft, Conceptualization, Resources. DZ: Writing – original draft, Writing – review & editing, Conceptualization.
